# Molecular targets for modulating the protein translation vital to proteostasis and neuron degeneration in Parkinson’s disease

**DOI:** 10.1186/s40035-019-0145-0

**Published:** 2019-02-04

**Authors:** Zhi Dong Zhou, Thevapriya Selvaratnam, Ji Chao Tristan Lee, Yin Xia Chao, Eng-King Tan

**Affiliations:** 1 Department of Research, National Neuroscience Institute, 11 Jalan Tan Tock Seng, Singapore, 308433 Singapore; 2 Department of Neurology, Singapore General Hospital, Outram Road, Singapore, 169608 Singapore; 3 Signature Research Program in Neuroscience and Behavioural Disorders, Duke-NUS Medical School Singapore, 8 College Road, Singapore, Singapore

**Keywords:** Molecular targets, Neuron degeneration, Parkinson’s disease, Protein aggregation, Protein translation, Proteostasis

## Abstract

Parkinson’s disease (PD) is the most common neurodegenerative movement disorder, which is characterized by the progressive loss of dopaminergic neurons in the Substantia Nigra pars compacta concomitant with Lewy body formation in affected brain areas. The detailed pathogenic mechanisms underlying the selective loss of dopaminergic neurons in PD are unclear, and no drugs or treatments have been developed to alleviate progressive dopaminergic neuron degeneration in PD. However, the formation of α-synuclein-positive protein aggregates in Lewy body has been identified as a common pathological feature of PD, possibly stemming from the consequence of protein misfolding and dysfunctional proteostasis. Proteostasis is the mechanism for maintaining protein homeostasis via modulation of protein translation, enhancement of chaperone capacity and the prompt clearance of misfolded protein by the ubiquitin proteasome system and autophagy. Deregulated protein translation and impaired capacities of chaperone or protein degradation can disturb proteostasis processes, leading to pathological protein aggregation and neurodegeneration in PD. In recent years, multiple molecular targets in the modulation of protein translation vital to proteostasis and dopaminergic neuron degeneration have been identified. The potential pathophysiological and therapeutic significance of these molecular targets to neurodegeneration in PD is highlighted.

## Background

Parkinson’s disease (PD) is the second most common neurodegenerative disorder with an incidence rate of 1% of the population over the age of 60 [1]. Furthermore, it is estimated that the number of individuals afflicted with PD will double by 2030 [2]. The pathological features of the disorder have been established as stemming from the selective and progressive degeneration of dopamine (DA) neurons in the Substantia Nigra pars compacta (SN) as well as the formation of protein inclusions known as Lewy bodies (LBs) in affected brain areas [3]. The progressive degeneration of DA neurons in the SN leads to a significant depletion of DA content in PD afflicted brains, which contribute to the onset of PD clinical symptoms, including tremors, akinesia, bradykinesia and stiffness [4]. Epidemiological studies show that PD arises as largely sporadic PD (SPD) in nature, and their exact underlying pathogenesis is still unclear. However, the onset of fewer familial forms of PD (FPD) can be induced by mutations or variations of a dozen or more genes, including α-synuclein (α-syn) [5], Parkin [6], PINK1 [7], DJ-1 [8], FBXO7 [9], CHCHD2 [10] and LRRK2 [11]. Currently, PD is still an incurable neurodegenerative disorder, and L-DOPA replacement therapy can transiently alleviate PD symptoms with no therapeutic effects on the progressive degeneration of DA neurons in PD patient brains.

**Fig. 1:**
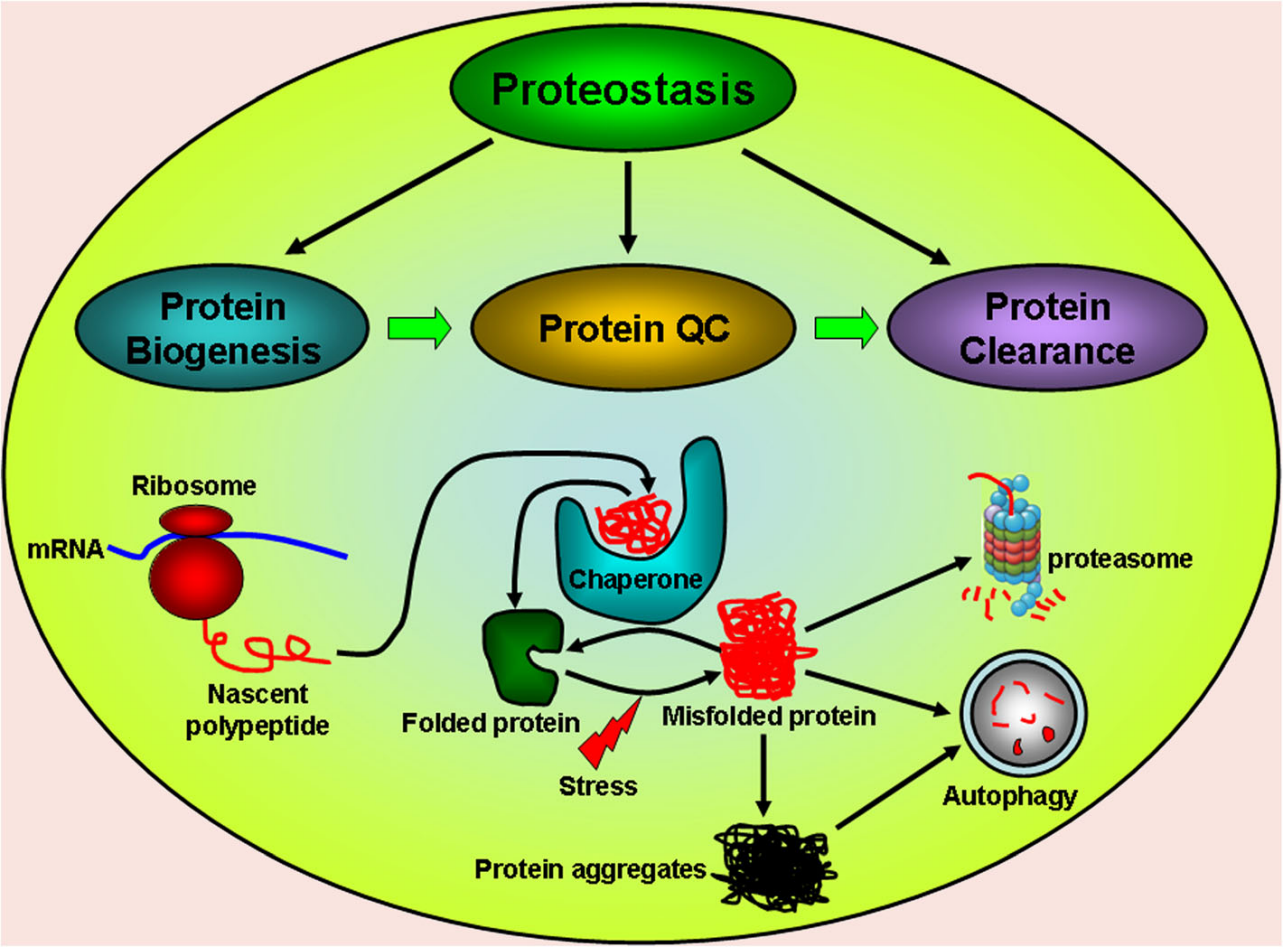
Molecular mechanisms for proteostasis maintenance Proteostasis can be maintained via three distinct and interlinked mechanisms, including the modulation of protein biogenesis, enhancement of chaperone capacity and prompt clearance of misfolded protein by UPS and autophagy. The ribosomal synthesis of nascent polypeptide is exquisitely modulated. The synthesized polypeptide can be folded into functional proteins with the assistance of chaperones. Chaperones can also function to refold stress-induced misfolded proteins. The misfolded protein can be cleared away by UPS and autophagy. However, the deregulated modulation of protein biogenesis and impairment of chaperone function, UPS and autophagy capacities can lead to disturbed proteostasis and protein aggregate formation

**Fig. 2:**
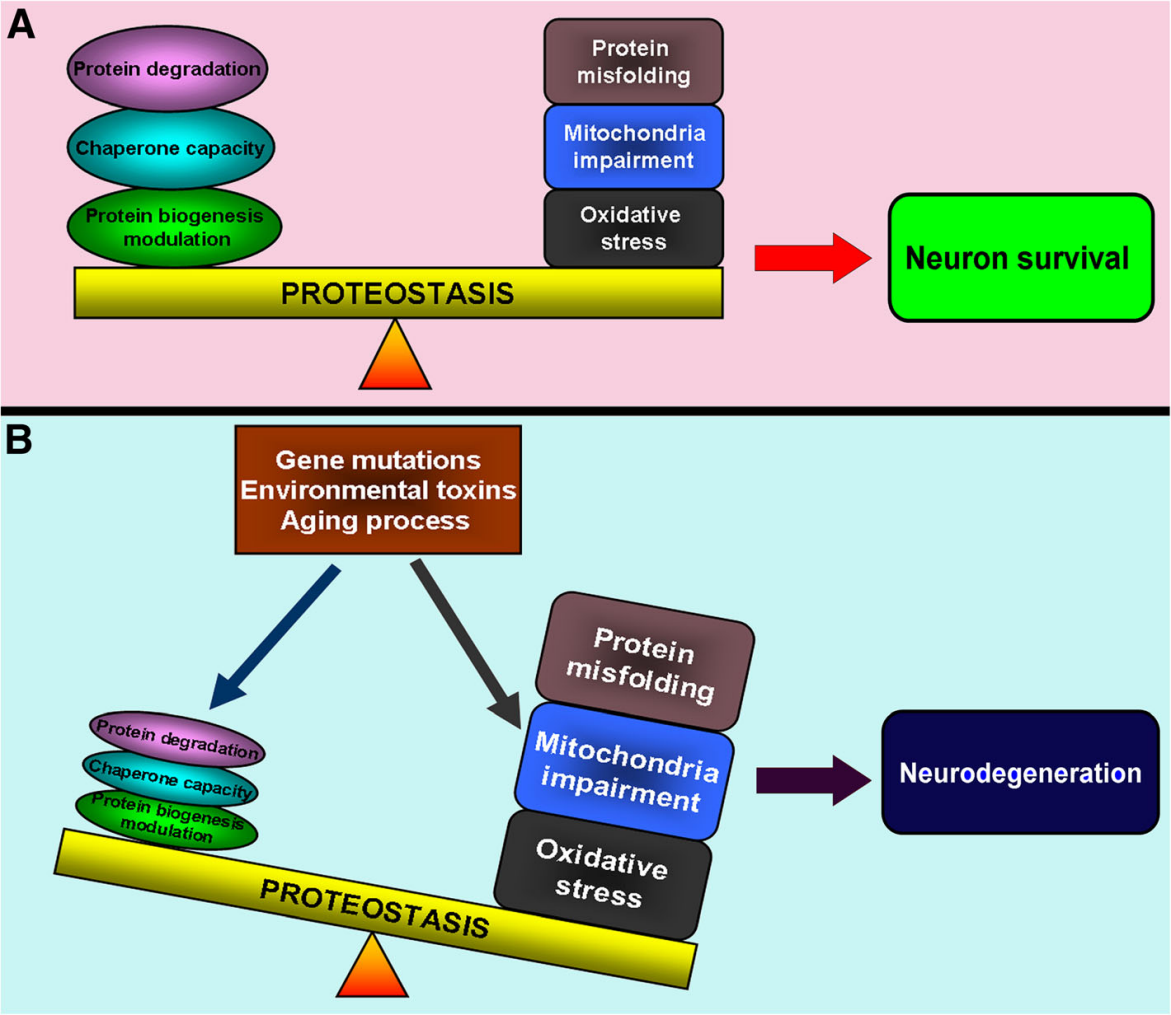
Balance and imbalance of proteostasis implicated in PD pathogenesis Under physiological conditions, the modulation of protein biogenesis, chaperone capacity and protein degradation can counteract against deleterious factors and stress challenge-induced protein misfolding and proteostasis dysfunction (**a**). Under pathological conditions, such as PD-associated gene mutations, environmental toxin challenges and pathological aging, the protective capacities of the proteostasis mechanisms are impaired, whereas stress-induced protein misfolding, mitochondria impairment and oxidative stress are aggravated. This can lead to the imbalance of proteostasis and protein aggregation, contributing to neurodegeneration in PD (**b**)

**Fig. 3:**
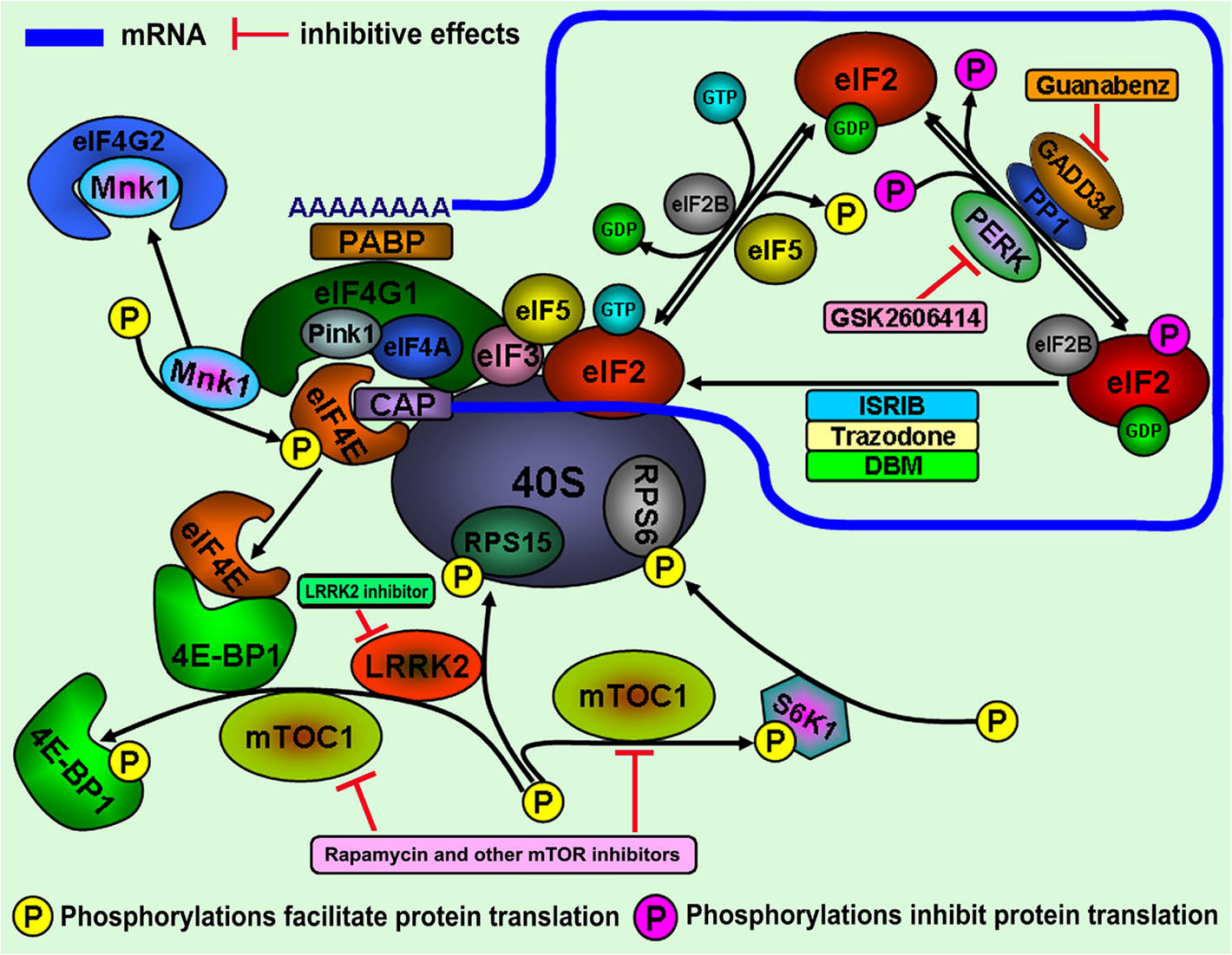
Molecular targets in the modulation of protein translation initiation implicated in proteostasis and PD pathogenesis and therapy Ribosomal protein biogenesis can be exquisitely modulated on multiple targets mainly through the modulation of functions of protein targets via phosphorylation and dephosphorylation by kinases and phosphatases, respectively. Multiple factors including eIF4G1, eIF4E, eIF4A, eIF3, eIF5, and eIF2 take part in the formation of the translation initiation complex, which is vital for initiation of protein translation. The kinase-induced phosphorylation of eIF4E, 4E-BP1, RPS15 and RPS6 will facilitate protein translation, which is supposed to be adverse to the maintenance of proteostasis under stress and implicated in PD pathogenesis. Mnk1 can phosphorylate eIF4E to enhance its binding with eIF4G1 to promote translation initiation, which can be abrogated by eIF4G2 chelation. However, the function of eIF4E can be inhibited by 4E-BP1 sequestration, which can be abrogated by LRRK2 and mTORC1 kinase-induced 4E-BP1 phosphorylation. LRRK2 kinase can also phosphorylate RPS15 to enhance protein translation, whereas mTORC1 kinase can phosphorylate S6K1. The phosphorylated S6K1 subsequently phosphorylates RPS6, which in turn promotes protein translation. LRRK2 and mTORC1 kinase inhibitors are supposed to have potential therapeutic effects against neurodegeneration in PD. On the other hand, the phosphorylation of eIF2α by PERK kinases can inhibit protein biogenesis. However, GADD34 can direct PP1 to dephosphorylate eIF2α, which can restore protein translation. GBZ can block GADD34 to promote eIF2α phosphorylation and arrest protein translation, whereas GSK2606414 can inhibit kinase-induced eIF2α phosphorylation to recover protein biogenesis. ISRIB, Trazodone and DBM can function downstream of eIF2α phosphorylation without influence on eIF2α phosphorylation to promote protein translation. However, all three FDA-approved drugs (GBZ, Trazodone and DBM) claim to have protective capacities against neurodegeneration in PD

One of the pathological features of PD is LB formation which are composed of multiple aggregated proteins in affected brain areas [12]. The formation of protein aggregates can be the pathological consequence of the disturbance and collapse of proteostasis [13]. Proteostasis refers to the maintenance of cellular protein homeostasis via multiple pathways that control the formation, folding, trafficking and clearance of proteins inside or outside the cell [14]. Proteostasis can be physiologically balanced by the upregulated levels and capabilities of chaperones, the enhanced efficiency in protein trafficking, the prompt clearance of misfolded proteins by ubiquitin proteasome system (UPS) and autophagy as well as the fine control of protein biogenesis [13] (Fig. 1). The maintenance of proteostasis is vital to many human physiological events including development, healthy aging, stress resistance and protection against pathogen invasion [14]. However, pathological factors, such as gene mutations, environmental toxins and pathological aging, can increase oxidative stress, impair mitochondria functions, aggravate protein misfolding and impair protective mechanisms, which will lead to disturbed and imbalanced proteostasis and cell demise (Fig. 2). Disturbed proteostasis inducing deleterious protein aggregation is relevant to the pathogenesis of various human disorders including cancer, obesity, PD and other human neurodegenerative disorders [15]. The primary modulation point to maintain the proteostasis is to exquisitely control the protein translation and biogenesis. This can be accomplished via kinase-induced phosphorylation and phosphatase-induced dephosphorylation of multiple ribosomal proteins, translation initiation factors and elongation factors indispensable for protein biogenesis (Fig. 3). The current review summarizes and discusses several identified molecular targets in the pathway for modulating protein translation vital to proteostasis and neuron degeneration in PD.

The protein translation process in eukaryotic cells includes three respective stages: translation initiation, elongation and termination [16]. The translation initiation process is the rate-determining step, which is controlled and coordinated by multiple eukaryotic initiation factors (eIFs) [17]. The eIFs play multiple roles in protein translation from activation of mRNA to the assembly of functional ribosomal subunits [18]. In principle, protein translation can be divided into two groups: cap-dependent and cap-independent mRNA translation [19]. In short, cap-dependent mRNA translation initiation occurs with the activation and circularization of mature mRNA and formation of a preinitiation complex (PIC) consisting of multiple eIFs and 40s ribosomal subunit (Fig. 3) [20, 21]. PIC can bind to the 5′-m^7^GpppX cap structure of mature mRNA to search for the starting codon in the mRNA 5′ untranslated region (5’UTR) for the initiation of translation [22]. Consequently, the 60S ribosomal subunit is recruited concomitantly with the release of eIFs, leading to the formation of the 80s ribosome complex for translation [23]. Alternatively, 3–5% of translation initiation can occur in a cap-independent manner, where ribosomes and eIFs are recruited to interact with the internal ribosomal entry site (IRES) or the cap-independent translation element (CITE) in mRNA to initiate translation [24]. After the initiation stage, nascent polypeptide chains can be generated and elongated during the translation elongation stage facilitated by eukaryotic elongation factor 2 (eEF2) [25]. eEF2 functions to mediate the positioning of the appropriate aminoacyl-tRNA to the acceptor site of the ribosome (A site), where the innate peptidyltransferase activity of the 80s ribosome will catalyze the formation of new peptide bonds between amino acids [26]. Furthermore, eEF2 promotes the translocation of the ribosome translation complex to the next codon in mRNA template to facilitate the elongation process [27, 28]. When the ribosome complex reaches the stop codon in mRNA template, multiple translation release factors (RFs) are recruited to release the new-born polypeptide from the ribosome, and protein translation is terminated [27, 28].

## Main text

### Eukaryotic initiation factor 2 (eIF2) as a molecular target in PD

eIF2 is the key factor for modulating protein translation at the translation initiation stage (Fig. 3) [29]. eIF2 is a heterotrimeric protein complex comprised of alpha, beta and gamma isoforms [30]. eIF2 is an essential initiation factor to interact with the initiator methionyl-tRNA (Met-tRNA_i_^Met^) and GTP to form an active ternary complex, which is essential for cap-dependent translation initiation [19]. Aided by other eIFs including eIF1, eIF1A and eIF3, this ternary complex interacts with the 40s ribosome to form PIC for translation initiation [19]. Subsequently, recruited eIF5 (a GTPase-activating protein) can induce eIF2 to hydrolyze GTP, leading to the dissociation of eIF2-GDP from the initiation complex and the beginning of protein translation after further recruitment of the 60S ribosomal subunit (Fig. 3) [19]. However, eIF2B, a Guanine nucleotide exchange factor (GEF), can function to exchange GDP in the inactive GDP-eIF2 complex with GTP to form the active GTP-eIF2, which can be used for a new round of translation initiation (Fig. 3) [19]. The alpha component of eIF2 protein complex has a phosphorylation site at Serine (S) 51 that can be phosphorylated by various stress-relevant kinases (e.g., PERK, PKR and GCN2) [31]. Phosphorylated eIF2 has high affinity to bind with eIF2B and inhibit the Guanine nucleotide exchange capacity of eIF2B, leading to formation of the inactive ternary complex (phosphorylated eIF2-GDP-eIF2B) (Fig. 3) [32]. The inactivated ternary complex will be incapable of being assembled into functional PIC to initiate protein translation [33]. Therefore, stress-induced eIF2α phosphorylation can lead to the transient shut down of global protein translation, thus providing a modulation mechanism for protein translation under stress [33]. However, stress induced eIF2α phosphorylation can also up-regulate specific gene expressions. The translation of ATF4, a key transcriptional factor to mediate endoplasmic reticulum (ER) unfolded protein response (erUPR), can be activated under stress induced eIF2α phosphorylation [34]. In mice liver, the translation of C/EBPα and C/EBPβ proteins was reported to be promoted by eIF2α phosphorylation [35]. Furthermore, eIF2α phosphorylation can activate cellular IRES elements to up-regulate IRES-mediated protein translation under a range of physiological circumstances [36]. The eIF2α phosphorylation can be counteracted by GADD34 to abrogate the stress-induced global translation arrestment via directing protein phosphatase 1 (PP1) to dephosphorylate the phosphorylated eIF2α as well as via its interaction with eIF2α to form a ternary complex to promote post-stress translation recovery (Fig. 3) [37].

Previous studies have demonstrated that eIF2 and its interacting proteins are essential for physiological brain function and development [18, 38]. The phosphorylation of eIF2α-induced shutdown of global protein translation can be the consequence of protein misfolding-induced erUPR [32]. The deregulation of erUPR and imbalance between phosphorylation and dephosphorylation of eIF2α is implicated in PD neuronal degeneration [32, 39]. The pathological accumulation of wild type (WT) and mutant α-syn can activate erUPR in PD brains [40, 41] . The accumulated α-syn in ER can bind with GRP78/BiP, leading to activation of erUPR through the PERK-dependent pathway [40, 42]. Furthermore the activation of erUPR will facilitate pathological α-syn aggregation [41]. Similarly, the accumulated tau protein in ER can impair ER-associated degradation (ERAD), leading to activation of erUPR and subsequent pathological phosphorylation of Tau protein [43]. The phosphorylated PERK and eIF2α have been detected in dopaminergic neurons in the SN of PD patients but not in healthy control cases [44]. The deregulated erUPR pathway and eIF2α phosphorylation can also be observed in peripheral blood mononuclear cells (PBMCs) of SPD and FPD patients [45]. Furthermore, eIF2α has been identified as a therapeutic target for PD [44]. The PERK kinase inhibitor GSK2606414 is demonstrated to prevent neuronal death in PINK1 and Parkin mutant flies [46]. Most recent findings demonstrate the neuroprotective capacity of GSK2606414 against PD-inducing neurotoxin-induced DA neuronal degeneration in a mouse PD model [47]. Although GSK2606414 is not suitable for applications to human PD patients due to its pancreatic toxicity [47], these findings indicate that targeting erUPR pathway and eIF2α phosphorylation hold promise towards the prevention of neurodegeneration in PD. A second compound, integrated stress response inhibitor (ISRIB) with the capacity to bind to eIF2B to activate its GEF activity under eIF2α phosphorylation [48], has been demonstrated to delay neurodegeneration in a prion mouse model [49]. However, the insoluble nature of ISRIB makes it difficult to be used in human patients [50]. In 2017, two FDA-approved drugs (Trazodone hydrochloride (Trazodone) and dibenzoylmethane (DBM)) with the capability to reverse eIF2α phosphorylation-induced protein translation arrestment and protect against in vivo neuron degeneration were identified from a phenotypic screening study [51]. DBM has displayed neuroprotective functions in both in vitro and in vivo PD models [52]. However, in 1998, a 74-year-old woman with depression symptoms after losing her sister was prescribed Trazodone to improve her mood [53]. Just several months after Trazodone usage, she began exhibiting Parkinsonism symptoms [53]. This was not an isolated case of Trazodone-induced motor issues after periodic usage of Trazodone [54]. Thus far, the pharmacological targets of Trazodone and DBM are still largely unknown and caution needs to be taken when these drugs are prescribed to PD patients. The ISRIB Trazodone and DBM can alleviate the eIF2α phosphorylation-induced protein translation arrestment without influencing the levels of phosphorylated eIF2α, suggesting that they function downstream of eIF2α phosphorylation (Fig. 3**)** [50].

On the other hand, Guanabenz (GBZ), a FDA-approved antihypertensive drug, is identified to be neuroprotective with capability to inhibit GADD34, leading to subsequent promotion of eIF2α phosphorylation, protein translation arrestment and maintenance of proteostasis [55–59]. GBZ can enhance eIF2α phosphorylation and protect against stress induced DA neuron degeneration in various PD models in an ATF4- and Parkin-dependent manner [60]. Recently Sephin1, a GBZ derivative with specific GADD34 inhibition capability but lack of α2-adrenergic agonist activity, is developed to protect against neuron degeneration relevant to erUPR induced by accumulation of misfolded proteins [61]). However, findings from a recent study challenge the view that GBZ and Sephin1 can restore proteostasis via interfering with the dephosphorylation of phosphorylated eIF2α protein [62]. GBZ can function independent on modulation of eIF2α phosphorylation [63–65]. The GBZ has anti-inflammatory effects mediated by eIF2α-dependent and eIF2α-independent mechanisms [63]. Furthermore GBZ can specifically inhibit the protein folding activity of the ribosome (PFAR), which is implicated in the pathogenesis of human neuron degenerative diseases [65]. The PFAR is referred to the function of rRNA of the large ribosomal subunit to facilitate protein folding [65]. GBZ can inhibit PFAR by competition with protein substrates for the common binding sites on the domain V of rRNA [64, 65]. The neuroprotective mechanisms of GBZ and Sephin1 dependent on modulation of eIF2α phosphorylation and should be paid more attention and warrants further investigations.

Thus far, the three FDA-approved drugs (GBZ, Trazodone and DBM) exert opposing influences on eIF2α phosphorylation-induced alterations of global protein translation. GBZ promotes the phosphorylation of eIF2α and the inhibition of protein translation. Therefore, GBZ may relieve the stress-induced accumulation of misfolded proteins, protein aggregation, proteostasis disturbance and cell stress, leading to neuroprotective effects. Conversely, Trazodone and DBM work to inhibit the eIF2α phosphorylation-induced protein translation arrestment, leading to neuroprotection as demonstrated in various in vitro and in vivo PD models [52]. They have opposing effects on eIF2α phosphorylation-modulated protein translation, but all drugs have been claimed to be neuroprotective. These findings are interesting. The accumulated misfolded protein and protein aggregation can lead to the imbalance of proteostasis, which can be a stress challenge to cells (Fig. 2) [66]. Therefore, the arrestment of protein translation induced by eIF2α phosphorylation under stress can help cells alleviate protein misfolding and aggregation. This mechanism may account for the GBZ-induced neuroprotective effects. However, the persistent activation of erUPR and prolonged arrestment of protein translation can also be detrimental to cells [67]. Therefore, the Trazodone- and DBM-induced release of arrested protein translation under eIF2α phosphorylation can rescue neurons from prolonged and persistent erUPR-induced neurodegeneration. This mechanism may also account for GSK2606414- and ISRIB-induced neuroprotective effects. However, releasing the protein misfolding-induced arrestment of protein translation at an earlier stage may aggravate the deleterious protein aggregation and proteostasis disturbance, which can trigger neuron degeneration. This may account for the Trazodone-induced motor issues and Parkinsonism symptoms in selected individual patient cases. Further works are needed on drugs targeting erUPR pathway and eIF2α phosphorylation-induced modulation of protein translation and proteostasis maintenance. Caution should be taken when these drugs are applied to PD patients with different etiologies and distinct disease stages of PD.

### Eukaryotic initiation factor 4G1 (eIF4G1) as a molecular target in PD

Eukaryotic initiation factor 4F (eIF4F) is a complex of multiple initiation factors, including the eIF4A and its cofactors eIF4B, eIF4E and eIF4G1 (Fig. 3) [68]. The eIF4F complex binds to the 5′-m^7^GpppX cap structure of the mRNA template while the poly-A binding protein (PABP) can bind to the poly-A tail of the mRNA, resulting in the circularization of the mRNA (Fig. 3) [69]. The eIF4F and mRNA cap complex then initiates protein translation by recruiting the PIC to the cap complex [70]. In the eIF4F complex, eIF4G1 acts as the main scaffold, binding to eIF4E, eIF4A and eIF3e as well as other molecules, such as PABP and the ribosome subunit (Fig. 3) [70]. When the availability of eIF4E is limited, eIF4G1 can initiate cap-independent translation through the formation of eIF4G1/eIF4A complexes and the recruitment of IRES-containing mRNA [71]. In humans, the overexpression of eIF4G1 is implicated in cancer and oncogenesis [72], whereas In yeast and nematodes eIF4G1 is found to be vital to organism development, wherein knock out of eIF4G1 is detrimental [73]. In addition to the down regulation of overall translation, inhibition of eIF4G1 alters the stoichiometry of mRNA translation supporting expression of genes vital to stress response in *C. elegans* [74]. The inhibition of eIF4G1 expression in adult stage extends the lifespan of *C. elegans* [74].

Recently, mutations of eIF4G1 were found to be linked to the pathogenesis of DA neuron degeneration in FPD. A genome-wide analysis study (GWAS) reported by Chartier-Harlin revealed the presence of eIF4G1 missense mutations p.Ala502Val (A502V) and p.Arg1205His (R1205H) in a French family and seven other PD-afflicted families from different countries but was absent in 4050 healthy controls [75]. Whole-genome sequencing among Americans also verified the presence of the R1205H eIF4G1 mutation in FPD patients [76]. Other variants of eIF4G1 identified in FPD include p.Gly686Cys (G686C), p.Ser1164Arg (S1164R) and p.Arg1197Trp (R1197W) [77–81]. However, follow-up studies carried out in a larger European cohort have questioned the causality of the R1205H eIF4G1 variant with PD onset [77–81]. Other novel but rare potential PD-linked eIF4G1 variants identified in these studies include p.Thr318Ile, p.Val541Gly, p.Gly698Ala, p.Pro486Ser [79], p.A425V, p.A428M, p.V541G, p.P486S, indels pE525del, pG466_A468del [76] and E462delInsGK [78]. Similar to the R1205H mutation, the eIF4G1 variants p.M432 V, p.A550P, p.P1229A, and p.L1233P are detected in both control and PD cases [78]. The E462delInsGK variant was observed to be segregated in two PD siblings [78]. Moreover, studies in other ethnic groups reveal the eIF4G1 variants to be extremely rare in PD patients and negative for the prevalent eIF4G1 variants in PD patients of Asia [82–84], South Africa [85] and Greek ethnicities [86]. Collectively, these conflicting reports suggest that the mutations in the eIF4G1 gene are likely to be benign polymorphisms or are linked to FPD with an extremely rare prevalence rate of less than 1% of PD incidence worldwide [76, 80].

Nevertheless, in vitro studies suggest the potential pathological role of eIF4G1 mutants in PD pathogenesis. It is identified that the eIF4G1 A502V variant obstructs the binding of eIF4G1 to eIF4E, thereby interfering with the recruitment of mRNA to the ribosome and subsequent cap-dependent translation [75]. Similarly, the eIF4G R1205H variant hinders the binding of eIF4G1 to eIF3, affecting interactions among mRNA, eIF4F cap binding complex and 40s ribosomal subunit [75]. Apart from this, the eIF4G1 gene is revealed to be genetically and functionally associated with other PD genes, further elaborating its potential pathological roles in PD. The overexpression of eIF4G1 or TIF4631 (the yeast homolog of eIF4G1) was found to alleviate α-syn toxicity in a yeast PD model [87]. However, overexpression of the R1205H mutant eIF4G1 impaired its capacity to inhibit α-syn-induced toxicity [87]. Another PD-relevant gene pathologically linked to eIF4G1 gene is VPS35, a protein associated with the retrograde transport of proteins from the endosome to the trans-Golgi network. Mutations of VPS35 have been identified to be linked to autosomal dominant PD [88]. It was demonstrated that, when protein translation was influenced by the upregulated level of TIF4631, yeast cells lacking VPS35 experienced aggravated toxicity. This toxicity can only be abated by the introduction of WT VPS35 rather than the PD-linked p.D620N mutant VPS35. However, the loss of TIF4631 and VPS35 genes in yeast models did not induce any lethality. This finding indicates that the deregulation of eIFG41 function under stressed conditions, such as proteotoxic stress induced by VPS35 deletion, can be deleterious. It is also demonstrated that PINK1 may interact with eIF4G1 and eIF4A in the initiation complex in an RNA-dependent manner (Fig. 3) [89]. The PD-linked G309D mutation in PINK1 hindered the interactions between PINK1 and eIF4G1. The inhibition of eIF4G1 in PINK1 mutant flies aggravated the neuromuscular degenerative phenotype [89]. Overexpression of eIF4G1 or calpastatin (an inhibitor of protease calpain, which cleaves eIF4G1) can lead to elevated levels of protein synthesis and improved viability in hippocampal CA1 neurons [88]. Although the pathological association of eIF4G1 as a PD gene with DA neuron degeneration in PD is still controversial, its significant roles in protein translation and its mutual crosstalk with other PD genes make it a potential molecular target in the proteostasis pathway for future studies in PD.

### Eukaryotic initiation factor 4E (eIF4E) and eIF4E-binding protein 1 (4E-BP1) pathway in PD

eIF4E is the initiation factor for determining whether the cap-dependent or IRES-mediated cap-independent protein translation will be initiated [90]. eIF4E directly binds to the cap structure of mRNA to facilitate the formation of the eIF4F complex on the mRNA cap structure, leading to the initiation of cap-dependent protein translation (Fig. 3) [91]. The integrity of protein structure and function of eIF4E determines the rate of global protein translation [92]. As a result, the tight control of the level and function of eIF4E is a necessity for the exquisite modulation of protein translation and proteostasis maintenance [92]. Regulation of the function of eIF4E can be a complicated model of modulation by kinase phosphorylation and its binding partners [68, 92]. eIF4E can be directly phosphorylated at S209 by eIF4E kinases, such as MAPK-activated protein kinase 1 (Mnk1), to enable its interaction with other initiation factors to form a stable eIF4F complex and enhance translation initiation [93]. eIF4E availability to protein translation initiation can also be modulated by its binding partners, mainly 4E-BP1 and eIF4G1 [94]. When cells are in a state conducive for global protein translation, eIF4G1 can bind to the dorsal surface of eIF4E via a recognition motif opposite to the cap binding pocket, promoting the interaction of eIF4E with the mRNA template and the initiation of translation [95]. eIF4G1 can also function as a scaffold to provide a docking site for Mnk1 to mediate the phosphorylation of eIF4E to enhance its function [96]. However, the paralog of eIF4G1, eIF4G2 (also known as P97), can interact with and sequester Mnk1 away from eIF4E, thereby inhibiting Mnk1-induced eIF4E phosphorylation and protein translation [96]. The influence of eIF4E function by 4E-BP1 is also vital to the modulation of protein translation [97]. The binding of 4E-BP1 with eIF4E will sequester eIF4E away from the assembly of the eIF4F initiation complex, thus blocking protein translation [95, 98]. The 4E-BP1-induced modulation of the protein translation can be affected by levels of 4E-BP1 and eIF4E in cells [97]. When the levels of intracellular eIF4E exceed the levels of 4E-BP1 in a dynamic cellular environment, the inhibition of protein translation by 4E-BP1 becomes ineffective [71]. Furthermore, kinase-induced 4E-BP1 phosphorylation can abrogate its binding with eIF4E, leading to the facilitation of protein translation [99]. 4E-BP1 can be phosphorylated and modulated by the mTOR signaling pathway and LRRK2 kinases [100]. Hyper-phosphorylated 4E-BP1 will be dissociated from eIF4E, leading to enhanced cap-dependent protein translation [71]. However, in the absence of growth factors and/or cellular stress, 4E-BP1 remains unphosphorylated, allowing it to competitively sequester the eIF4E, thereby inhibiting the translation initiation mechanism [101].

Disturbance of the eIF4E and 4E-BP1 pathway can be disease related. 4E-BP1’s function is understood to be neuroprotective, whereas elevated levels of eIF4E inducing translation facilitation can be pathological. It was found that the deregulated translation induced by either the upregulation of eIF4E or knock-out of 4E-BP1 is relevant to the onset of autism spectrum disorder in mice [102]. The upregulated expression of eIF4E can contribute to tumor formation [103]. Recent studies have implicated the relevance of the eIF4E and 4E-BP1 pathway in PD pathogenesis [32]. The levels of eIF4E can be modulated via ubiquitin-mediated proteasomal degradation [104]. Parkin, a PD-related E3 ubiquitin ligase, was found to interact with eIF4E and colocalize in developing oocytes [105]. In Drosophila ovarian models with the Parkin P23 mutant, the level of eIF4E is elevated [105]. Furthermore, suppression of eIF4E can rescue the observed fertility and developmental defects in the viability and size in Parkin P23 mutant Drosophila pupae [105]. Therefore, Parkin may function as the E3 ubiquitin ligase to promote the degradation of eIF4E by UPS. The Parkin mutation-induced impairment of Parkin E3 ligase activity may lead to the upregulation of eIF4E levels, which can be deleterious to proteostasis maintenance and DA neuron cell viability under stress. Furthermore, the eIF4E and 4E-BP1 pathway can be modulated by LRRK2 kinases [106]. LRRK2 is found to directly phosphorylate 4E-BP1 at the site Threonine (T) 37/T46 both in vivo and in vitro, leading to subsequent hyperphosphorylation of 4E-BP1 at T70 and S65 by LRRK2 or other protein kinases [106]. The phosphorylation of 4E-BP1 by the LRRK2 kinase promotes the dissociation of eIF4E from 4E-BP1, leading to enhanced eIF4E functions, accelerated protein translation and disturbed proteostasis under stress [106]. The PD-linked mutant LRRK2, such as G2019S LRRK2 mutant with increased LRRK2 kinase activity, can induce hyperphosphorylation of 4E-BP1 and deregulated protein translation, which can be relevant to LRRK2 mutation-induced DA neuron degeneration in PD [106]. The neuroprotective roles of 4E-BP1 can be evidenced in multiple PD models. The overexpression of 4E-BP1 was suggested to alleviate the Drosophila PINK1 mutant phenotype via upregulation of the cap-independent translation of various stress-related genes, including anti-oxidant genes [107]. The loss of Drosophila LRRK2-induced hypo-phosphorylation of 4E-BP1 can contribute to the protection of DA neurons and the alleviation of PD-like symptoms in Parkin/PINK1 mutant fly PD models [107]. The overexpression of Thor, the Drosophila ortholog of mammalian 4E-BP1, in Parkin loss-of-function or PINK1 mutant Drosophila can suppress DA neuron degeneration and alleviate the PD-like phenotype in these flies [107]. Furthermore, the overexpression of 4E-BP1 can also rescue PD phenotypes in CHCHD2 loss-of-function Drosophila PD model [108]. Thus far, accumulated evidence implicates the important functional balance of eIF4E and 4E-BP1 in the modulation of protein translation, which is vital to proteostasis maintenance and neuron survival under stress. Therefore, drugs or strategies targeting the eIF4E and 4E-BP1 pathway may have therapeutic significance to protect against neuron degeneration in PD and other neurodegenerative diseases.

### Ribosomal protein S15 (RPS15) as a molecular target in PD

The human ribosomal protein RPS15 is a component of the 40S ribosome subunit and plays a central role in ribosome biogenesis and protein translation [109]. RPS15 is shown to stimulate both cap-dependent and cap-independent protein translation [110]. It was reported that RPS15 can function to promote the export of pre-40S particles from the nucleus to the cytosol [111]. Mutations in RPS15 were found to be attributed to 10–20% of aggressive chronic lymphocytic leukemia [112]. The upregulated level of RPS15 was found to be connected to nasopharyngeal carcinoma with significant roles of RPS15 in the modulation of protein translation [113]. Recent findings have demonstrated that RPS15 can be the substrate of LRRK2 kinases, which is implicated in LRRK2 mutation-induced DA neuron degeneration in PD [114]. LRRK2 was demonstrated to phosphorylate RPS15 at T136 [114]. It was found that the pathogenic G2019S and I2020T mutant LRRK2 proteins promoted the phosphorylation of RPS15, contributing to the uncontrolled protein synthesis and subsequent DA neurotoxicity [114]. Thus, LRRK2 kinases may modulate protein translation via phosphorylation of both RPS15 and EF-4B1. The enhanced phosphorylation of RPS15 and EF-4B1 by LRRK2 mutants can promote global protein translation [110, 114]. Previous studies have demonstrated that endogenous DA can be a deleterious factor in DA neurons, as DA can be oxidized to generate toxic byproducts, inclusive of reactive oxygen species (ROS) and highly reactive DA quinones (DAQ) [115]. The toxic byproducts derived from DA oxidation can actively modify the function of proteins, leading to inactivation of proteins and protein misfolding and aggregation [115]. Therefore, the enhanced protein translation induced by PD-linked LRRK2 mutations in DA neurons may facilitate the accumulation of DA modified and misfolded proteins, which can be adverse to proteostasis maintenance and DA neuron viability. However, it was found that PD-linked R1441C and R1441G LRRK2 mutants cannot influence the phosphorylation stage of RPS15 [114]. Phospho-deficient RPS15 cannot rescue R1441C LRRK2 mutant-induced DA neurotoxicity [114]. Therefore, more work is needed to investigate the PD-linked LRRK2 mutation-induced deregulation of protein translation and disturbance of proteostasis significant to PD pathogenesis and therapy.

### Molecular targets in the mammalian target of rapamycin (mTOR) pathway

Recent findings have implicated the mTOR pathway and its deregulation in PD pathogenesis [114]. mTOR is an evolutionary conserved, ubiquitous S/T protein kinase belonging to a subgroup of kinases called phosphoinositide 3-kinase-related kinases (PIKKs) [116]. The physiological function of the mTOR pathway is critical to synaptic plasticity, learning and cortical development as well as neuronal survival [117, 118]. The mTOR protein interacts with other proteins and serves as the core component of two distinct protein complexes: mTORC1 (the Rapamycin-sensitive mTOR complex 1) and mTORC2 (the Rapamycin-insensitive mTOR complex 2) [119].

mTORC1 is composed of the mTOR protein, the regulatory-associated protein of mTOR (Raptor), the mammalian lethal with SEC13 protein 8 (mLST8) and the noncore components PRAS40 and DEPTOR proteins [120]. mTORC1 kinase can function to modulate protein translation via phosphorylation of its two downstream substrates, ribosomal protein S6 kinase beta-1 (S6K1) and 4E-BP1 in a dynamic cellular environment. Hyperactive mTORC1 signaling can lead to the phosphorylation of 4E-BP1 and the release of eIF4E for enhancement of cap-dependent protein translation. Activated mTORC1 can also phosphorylate and activate S6K1 at T389 to further facilitate translation initiation and elongation via S6K1-induced subsequent phosphorylation of the ribosomal protein S6 (RPS6), eIF4B and elongation factor eEF2K, respectively [25]. On the other hand, mTORC1 kinase can inhibit autophagy via phosphorylation of the Unc51-like kinase 1 (ULK1) to inhibit the formation of a macrocomplex (ULK1 / Atg13 / FIP20) which is vital for autophagosome formation and autophagy initiation [121]. The inhibition of autophagy by activated mTORC1 kinase will inhibit the clearance of misfolded protein, which further aggravates protein aggregation, proteostasis disturbance and DA neuron viability impairment [122]. Thus far, findings have indicated that the hyperactive mTORC1 pathway is implicated in DA neurodegeneration, whereas modulation of the mTORC1 pathway can be significant to therapy against DA neurodegeneration in PD [123]. Selective mTORC1 inhibitors, Rapamycin and its analogues, have demonstrated some neuroprotective capacity in various PD models [124, 125]. Rapamycin was found to mitigate the side effect of the anti-PD drug L-Dopa, such as dyskinesia, in a PD mouse model [124]. Moreover, Temisrolimus, a Rapamycin analogue, is found to ameliorate the behavioral deficits in an MPTP mouse PD model [126]. Other mTORC1 inhibitors, such as metformin, minocycline and celastrol, are found to regulate protein translation via modulation of the mTORC1 kinase activity and contribute to improved proteostasis maintenance and DA neuron survival [127–129].

mTORC1 signaling was also implicated in genetic factor-induced DA neurodegeneration in PD. The mTORC1 kinase-induced activation of S6K aggravates the fly PD phenotype in a mutant PINK1 fly model, which can be rescued by WT Parkin [130]. However, down-regulation of protein translation by the knockdown of S6K, RPS6 or ribosomal protein 9 (RPS9) can rescue PINK1 mutant fly phenotypes, supporting the pathological link of the mTORC1 pathway with neurodegeneration in FPD [130]. In a study wherein hypoxia was induced, the loss of PINK1 was found to disrupt the dephosphorylation of 4E-BP1, leading to facilitated protein translation [131]. These findings have indicated the potential functional crosstalk between the mTORC1 and PINK1-Parkin pathways with relevance to protein translation modulation and DA neuron degeneration in PD [130]. LRRK2 was also shown to have crosstalk with the mTOR pathways via phosphorylation of 4E-BP1 and Akt [132]. A recent pilot screening-based preclinical study has identified new pharmacological agents with mTORC1 kinase inhibition capability to modulate protein translation and protect DA neurons in a DJ-1β mutant PD fly model [133]. New potent and capable neuroprotective mTORC1 inhibitors may be developed in the near future to treat progressive DA neuron degeneration in SPD as well as in FPD.

mTORC2 is composed of the mTOR protein, the Rapamycin-insensitive companion of mTOR (RICTOR), MLST8, and mammalian stress-activated protein kinase interacting protein 1 (mSIN1) [134]. mTORC2 play roles in the modulation of cell metabolism, motility, survival and proliferation [122]. Inhibition of mTORC2 will impair cell proliferation, which is implicated in cancer therapy [135, 136]. Akt is a downstream target of mTORC2 and is vital to cell viability and proliferation [122]. Multiple studies demonstrate that Akt/Akt1 can be a substrate of LRRK2 kinase and that the kinase activity of Akt can be abrogated by PD-associated LRRK2 mutations [137]. Therefore, the impairment of proliferative mTORC2-Akt pathway signaling by PD-linked LRRK2 mutants may also contribute to LRRK2 mutation-induced DA neuron degeneration in PD.

## Conclusions

The maintenance of cell proteostasis is vital to many physiological events, and disturbance of proteostasis can be pathologically significant for neurodegeneration in PD and other neurodegenerative disorders. This can be indicative through the formation of featured protein aggregates in affected patient brains with PD and other neurodegenerative diseases. Proteostasis can be maintained by modulation of protein translation, enhancement of chaperone capacity and protein clearance via UPS and autophagy. The modulation of protein translation to maintain proteostasis is the primary mechanism for cells to cope with stress-induced challenges. Previous findings have shown that the facilitated protein translation can be either adverse or advantageous to neuron survival under different scenarios [138, 139]. Similarly, inhibition of protein translation has been identified to be either protective or detrimental to cells [67]. The accumulation of misfolded proteins in the ER will activate erUPR and enhance phosphorylation of eIF2α protein [140]. The phosphorylated eIF2α can suppress global protein translation, which can help cells cope with protein misfolding-induced cell degeneration [140]. However, severe or prolonged erUPR can be deleterious to cells [141]. Prolonged inhibition of global protein translation can lead to apoptosis, which is a promising therapeutic strategy for cancer therapy [67]. However, the inhibition of protein translation is suggested to be neuroprotective in PD models [32]. The translation inhibition by acute exposure to cycloheximide is identified to inhibit hypertonicity-induced aggregation of polyglutamine and endogenous α-syn in *C elegans* [142]. Thus far, several FDA-approved drugs (GBZ, Trazodone and DBM) targeting eIF2α phosphorylation for inducing the arrest or facilitation of protein translation are shown to be neuroprotective against DA neuron degeneration in different PD models [32, 143]. GBZ can inhibit GADD34 to enhance eIF2α phosphorylation, contributing to the arrest of protein translation [144]. However, Trazodone and DBM can abrogate eIF2α phosphorylation-induced translation arrest and facilitate protein translation [51]. They have opposing impacts on eIF2α phosphorylation and protein translation, but all of these drugs are identified to have neuroprotective effects.

Similar situations can be identified in eIF4G1 and eIF4E-4E-BPs pathways. The mutant LRRK2 enhances the phosphorylation of 4E-BP1 to facilitate eIF4E-induced translation initiation and protein synthesis, which is suggested to be implicated in LRRK2 mutation-induced DA neuron degeneration in PD [106, 110]. The LRRK2 mutations can also phosphorylate the ribosomal RPS15 protein to facilitate protein translation [114]. These findings indicate that the accelerated protein biogenesis induced by LRRK2 mutations can be relevant to LRRK2 mutation-induced DA neuron degeneration. Other researchers have reported that the increased levels of 4E-BP1 to interact with and sequester eIF4E can be protective of DA neurons, suggesting that the deceleration of protein translation can promote cell survival and be neuroprotective [107, 145]. However, PD-linked eIF4G1 A502V and R1205H variants are found to disturb the protein translation initiation with the potential inhibition of protein translation, which is supposed to be relevant to eIF4G1 mutation-induced DA neuron degeneration in PD [75].

The dual influences of the opposing modulations of protein translation on cell viability can also be visualized in the mTORC1 signaling pathway. Inhibition of the mTORC1 pathway by Rapamycin has been demonstrated to be neuroprotective. However, overexpression of the WT mTOR protein or the constitutively active S6K1 to facilitate protein translation is found to protect against PD toxin-induced in vitro dopaminergic PC12 cell death [145]. mTORC1 can phosphorylate and inhibit ULK1 to suppress autophagy, which can be adverse to cell viability. However, it has recently been reported that ULK1 expression is upregulated to protect against MPP^+^-induced MN9D cell vulnerability via inhibition of mTOR kinase-induced T389 phosphorylation and activation of S6k1 [146]. Thus, ULK1 and mTOR kinase seem to form a complicated feedback loop with reciprocal modulation of their activities and functions.

Thus far, multiple molecular targets in pathways for modulating protein translation vital to proteostasis and cell viability have been identified. However, the facilitation or inhibition of protein translation can have complicated impacts on proteostasis and neuron survival [147]. Multiple and complicated factors may account for some inconsistent findings. Different in vitro and in vivo experimental models utilized and challenges with different stressors for different time periods and with different magnitudes may lead to distinct conclusions. For example, at a downstream erUPR stage, prolonged activation of erUPR can be lethal; therefore, the application of drugs to inhibit eIF2α phosphorylation and promote protein translation at a downstream erUPR stage can alleviate the erUPR-induced neurodegeneration. This mechanism may account for the GBZ-induced neuroprotection in some PD models. However, the administration of drugs inhibiting eIF2α phosphorylation at an earlier stage of erUPR may aggravate protein misfolding and aggregation, which can be deleterious to DA neuron survival. Such a mechanism may account for Trazodone-induced onset of PD symptoms in some patients. Currently, little is known about molecular targets and detailed molecular events in the modulation of protein translation and the subsequent impact on proteostasis and cell survival. More future works are warranted to improve our understanding of PD pathogenesis and contribute to the development of novel effective anti-PD drugs or therapies.

## Abbreviations

4E-BP1: eIF4E-binding protein 1
5’UTR: 5′ untranslated region
AD: Alzheimer’s disease
ALS: Amyotrophic lateral sclerosis
CITE: Cap-independent translation element
DA: Dopamine
DAQ: Dopamine quinone
DBM: Dibenzoylmethane
eEF2: eukaryotic elongation factor 2
eIF2: eukaryotic initiation factor 2
eIF4E: eukaryotic initiation factor 4E
eIF4F: eukaryotic initiation factor 4F
eIFs: eukaryotic initiation factors
ER: Endoplasmic reticulum
ERAD: ER-associated degradation
erUPR: ER unfolded protein response
FPD: Familial form of PD
GBZ: Guanabenz
GEF: Guanine nucleotide exchange factor
GWAS: Genome-wide analysis study
IRES: Internal ribosomal entry site
ISRIB: An integrated stress response inhibitor
LBs: Lewy bodies
Met-tRNA_i_^Met^: Initiator methionyl-tRNA
mLST8: mammalian lethal with SEC13 protein 8
mSIN1: mammalian stress-activated protein kinase interacting protein 1
mTOR: mammalian target of Rapamycin
mTORC1: Rapamycin-sensitive mTOR complex 1
mTORC2: Rapamycin-insensitive mTOR complex 2
PABP: Poly-A binding protein
PBMCs: Peripheral blood mononuclear cells
PD: Parkinson’s disease
PFAR: Protein folding activity of the ribosome
PIC: Preinitiation complex
PIKKs: Phosphoinositide 3-kinase-related kinases
PP1: Protein phosphatase 1
Raptor: Regulatory-associated protein of mTOR
RFs: Translation release factors
RICTOR: Rapamycin-insensitive companion of mTOR
ROS: Reactive oxygen species
RPS15: Ribosomal protein S15
RPS6: Ribosomal protein S6
S6K1: Ribosomal protein S6 kinase beta-1
SN: Substantia Nigra pars compacta
SPD: Sporadic form of Parkinson’s disease
TIF4631: yeast homolog of eIF4G1
ULK1: Unc51-like kinase 1
UPS: Ubiquitin-proteasome system
WT: Wild type
α-syn: α-synuclein
